# Weighted Gene Co-expression Network Analysis Identifies a Cancer-Associated Fibroblast Signature for Predicting Prognosis and Therapeutic Responses in Gastric Cancer

**DOI:** 10.3389/fmolb.2021.744677

**Published:** 2021-10-08

**Authors:** Hang Zheng, Heshu Liu, Huayu Li, Weidong Dou, Xin Wang

**Affiliations:** ^1^ Department of General Surgery, Peking University First Hospital, Peking University, Beijing, China; ^2^ Department of Oncology, Beijing Chaoyang Hospital, Capital Medical University, Beijing, China

**Keywords:** gastric cancer, cancer-associated fibroblasts, weighted gene co-expression network analysis, biomarker, prognosis, immunotherapy

## Abstract

**Background:** Cancer-associated fibroblasts (CAFs) are the most prominent cellular components in gastric cancer (GC) stroma that contribute to GC progression, treatment resistance, and immunosuppression. This study aimed at exploring stromal CAF-related factors and developing a CAF-related classifier for predicting prognosis and therapeutic effects in GC.

**Methods:** We downloaded mRNA expression and clinical information of 431 GC samples from Gene Expression Omnibus (GEO) and 330 GC samples from The Cancer Genome Atlas (TCGA) databases. CAF infiltrations were quantified by the estimate the proportion of immune and cancer cells (EPIC) method, and stromal scores were calculated via the Estimation of STromal and Immune cells in MAlignant Tumors using Expression data (ESTIMATE) algorithm. Stromal CAF-related genes were identified by weighted gene co-expression network analysis (WGCNA). A CAF risk signature was then developed using the univariate and least absolute shrinkage and selection operator method (LASSO) Cox regression model. We applied the Spearman test to determine the correlation among CAF risk score, CAF markers, and CAF infiltrations (estimated *via* EPIC, xCell, microenvironment cell populations-counter (MCP-counter), and Tumor Immune Dysfunction and Exclusion (TIDE) algorithms). The TIDE algorithm was further used to assess immunotherapy response. Gene set enrichment analysis (GSEA) was applied to clarify the molecular mechanisms.

**Results:** The 4-gene (COL8A1, SPOCK1, AEBP1, and TIMP2) prognostic CAF model was constructed. GC patients were classified into high– and low–CAF-risk groups in accordance with their median CAF risk score, and patients in the high–CAF-risk group had significant worse prognosis. Spearman correlation analyses revealed the CAF risk score was strongly and positively correlated with stromal and CAF infiltrations, and the four model genes also exhibited positive correlations with CAF markers. Furthermore, TIDE analysis revealed high–CAF-risk patients were less likely to respond to immunotherapy. GSEA revealed that epithelial–mesenchymal transition (EMT), TGF-β signaling, hypoxia, and angiogenesis gene sets were significantly enriched in high–CAF-risk group patients.

**Conclusion:** The present four-gene prognostic CAF signature was not only reliable for predicting prognosis but also competent to estimate clinical immunotherapy response for GC patients, which might provide significant clinical implications for guiding tailored anti-CAF therapy in combination with immunotherapy for GC patients.

## Introduction

Gastric cancer (GC) ranks fifth among the most common cancers and is the fourth leading cause of cancer-related mortality worldwide (Sung et al., 2021). Leaving aside improvements in gastroscopic screening and various treatment strategies, recurrence and metastasis remain the main causes of GC death, and the current therapeutic efficacy on recurrent and metastatic GC is still unsatisfactory (Lee et al., 2016; Thrift and El-Serag, 2020). GC tissues are composed of neoplastic cancer cells as well as the immune and stromal milieu where tumor cells are located, which is termed as tumor microenvironment (TME). Accumulating evidence indicated tumor stromal components in TME are critical for tumor growth and metastasis, immunosuppression, and drug resistance (Hanahan and Coussens, 2012; Quail and Joyce, 2013), which have embraced a spacious field of investigation.

As the most prominent cell type of tumor stroma, cancer-associated fibroblasts (CAFs) are crucial sources of growth factors and cytokines that promote tumor progression and migration (Kojima et al., 2010; Tommelein et al., 2015), stimulate epithelial–mesenchymal transition (EMT) (Wu et al., 2017; Fiori et al., 2019), and induce chemoresistance (Lotti et al., 2013; Li et al., 2016) and immunosuppression (Kraman et al., 2010; Monteran and Erez, 2019). CAFs are also capable of depositing and reorganizing the extracellular matrix (ECM), which serves as a thick physical barrier that supports tumor cell invasion and restrains the infiltrations of antitumor leukocytes, leading to tumor progression, immune evasion, and therapy resistance (Ma et al., 2016; Lakins et al., 2018; Kaur et al., 2019; Gamradt et al., 2021). Thus, targeting CAF-mediated immunosuppressive stromal microenvironment in combination with immunotherapy could promisingly ameliorate the response to immune checkpoint inhibitors. For instance, exhaustion of fibroblast activation protein (FAP)–positive CAFs in murine models led to increased CD8^+^ T cell infiltrations and decreased macrophages proportions (Duperret et al., 2018), and therapeutic effects of anti-CTLA4 and anti-PD-1 were consequently enhanced (Feig et al., 2013). Unfortunately, FAP-based drugs against CAFs failed to pass Phase II trials owing to the unsatisfactory clinical response in metastatic colorectal cancer patients (Hofheinz et al., 2003; Narra et al., 2007), and such a CAF inhibitory strategy is currently lacking in GC treatment. In this regard, it is imperative to explore stromal CAF-related factors in GC.

Weighted gene co-expression network analysis (WGCNA) is a systematic bioinformatics algorithm that is competent to incorporate highly and coordinately expressed genes into several gene modules and investigate the module’s relationships with the phenotype of interest (Langfelder and Horvath, 2008). WGCNA has been successfully applied for identifying CAF markers (Liu et al., 2021a; Liu et al., 2021b). So far, CAF and stromal infiltrations have not been subjected to WGCNA analysis in GC. In this study, for the first time, WGCNA was employed simultaneously on two transcriptome datasets collected from publicly available Gene Expression Omnibus (GEO) and The Cancer Genome Atlas (TCGA) databases. We detected hub modules that were most correlated with stromal CAF infiltrations. Then, by applying univariate and Least Absolute Shrinkage and Selection Operator (LASSO) Cox regression analyses, we identified COL8A1, SPOCK1, AEBP1, and TIMP2 as prognostic CAF markers and constructed the four-gene CAF signature capable of predicting prognosis and therapeutic responses in GC. Our results hint that the CAF model might be a novel anti-CAF therapeutic approach in GC.

## Materials and Methods

### Data Acquisition and Preprocessing

The fragments per kilobase of transcript per million mapped reads (FPKM) format RNA-seq data and corresponding prognostic data (follow-up time more than 30 days) of 330 TCGA stomach adenocarcinoma (TCGA-STAD) samples were downloaded through UCSC Xena browser (GDC hub) (https://gdc.xenahubs.net) (Goldman et al., 2020). The normalized FPKM values were converted to transcripts per million (TPM) and log2(TPM+1) transformed (Wagner et al., 2012). We also obtained normalized expression data and clinical information of 431 GC samples in GSE84437 from the GEO database (Yoon et al., 2020). The highest value was reserved if one gene matched multiple probes.

### CAF Infiltration Estimation and Stromal Score Calculation

CAF abundances were separately estimated *via* four methods: cell-type deconvolution (constrained least square optimization)–based Estimate the Proportion of Immune and Cancer cells (EPIC) algorithm (Racle et al., 2017), gene signature enrichment–based xCell algorithm (Aran et al., 2017), marker genes expressions–based microenvironment cell populations-counter (MCP-counter) (Becht et al., 2016), and Tumor Immune Dysfunction and Exclusion (TIDE) algorithms (Jiang et al., 2018). The first three methods were achieved *via* a deconvolute() function of immunedeconv R package (version 2.0.3) (Sturm et al., 2020), and the TIDE method was implemented through http://tide.dfci.harvard.edu/. In addition, the Estimation of STromal and Immune cells in MAlignant Tumor tissues using Expression data (ESTIMATE) algorithm was applied to calculate the stromal score *via* estimate R package (version 1.0.13), which indicates the stromal infiltrating levels of each tumor sample (Yoshihara et al., 2013).

### CAF and Stromal Co-expression Network Constructions

Co-expression networks and hub genes that targeted CAF infiltrations as well as stromal scores were constructed and detected *via* WGCNA R package (version 1.68) (Langfelder and Horvath, 2008). Genes with the top 5,000 of median absolute deviation (MAD) were first chosen as the input genes for network constructions in both TCGA-STAD and GSE84437 cohorts. Then, the Pearson’s correlation similarity matrix between any gene pairs was calculated (s_ij_, where ij represents pair-wise genes) and increased to soft-thresholding power β (s_ij_^β^) based on the scale-free topology network criterion. Subsequently, the adjacency matrix was clustered using topological overlap measure (TOM) and dissimilarity (1-TOM) between genes, and we conducted a dynamic tree cut algorithm on the dendrogram for gene module identifications with minimum gene numbers as 30 in each module. Each module expression’s first principal component was summarized as module eigengenes (MEs), the Pearson’s correlations between MEs and EPIC-quantified CAF infiltrations as well as the stromal score were evaluated, and the most correlated module was picked for further analysis. Then, we measured gene significance (GS) for the traits and module membership (MM indicates the correlation between ME and gene expression) of individual genes in the identified hub module, and hub genes were filtered out under the strict criteria of GS > 0.4 and MM > 0.8. Finally, the overlapping hub genes between TCGA-STAD and GSE84437 cohorts constituted the final hub genes.

### Gene Ontology (GO) and the Kyoto Encyclopedia of Genes and Genomes (KEGG) Analyses

GO and KEGG pathway enrichment analyses were performed on the final hub genes to identify the biological functions (including biological processes (BPs), molecular functions (MFs), and cellular components (CCs)) and pathways through clusterProfiler R package (version 3.14.3) (Yu et al., 2012). *p* < 0.05 was considered statistically enriched.

### Prognostic Model Construction and Validation

The GSE84437 cohort was selected for CAF risk model construction owing to its larger sample size, while 330 cases from TCGA-STAD were assigned to the validation cohort. The univariate Cox regression model was performed to identify prognostic stromal CAF hub genes on overall survival (OS); genes with *p* < 0.05 were subsequently put into LASSO Cox regression analysis with 1,000 iterations for gene reduction *via* glmnet R package (Simon et al., 2011). Then, the CAF risk model was constructed as follows: CAF risk score = Ʃ (β_i_ * Exp_i_), where β_i_ refers to the LASSO coefficient of *i*th gene, and Exp_i_ represents the *i*th gene’s expression value. GC patients were classified into high– and low–CAF-risk groups based on their median CAF risk scores, and the OS difference between two groups was estimated *via* Kaplan–Meier curves and the log-rank test. Similarly, the CAF risk model was validated in the TCGA-STAD cohort.

### CAF Markers Collections and Correlation Analysis

CAF specific and nonspecific markers were collected from published literature (Gascard and Tlsty, 2016; Han et al., 2020). To ensure the reliability of our CAF model markers in GC, we analyzed the Spearman’s correlations between the CAF risk score and stromal score as well as multi-estimated CAF infiltrations (EPIC, xCell, MCP-counter, and TIDE). Correlations between CAF model genes and published CAF markers were also analyzed on both TCGA-STAD and GSE84437 cohorts.

### Chemotherapy and Immunotherapy Response Predictions

Based on the largest publicly attainable pharmacogenomics database, Genomics of Drug Sensitivity in Cancer (GDSC) (https://www.cancerrxgene.org/) (Yang et al., 2013), half-maximal inhibitory concentration (IC50) values of common drugs (bleomycin, lapatinib, paclitaxel, camptothecin, cisplatin, docetaxel, methotrexate, and sunitinib) in each GC sample were estimated based on the transcriptome data by ridge regression with ten-fold cross-validation in pRRophetic R package (version 0.5) (Geeleher et al., 2014a; Geeleher et al., 2014b). Subsequently, the TIDE (http://tide.dfci.harvard.edu/) online algorithm was adopted for immune checkpoint blockade therapy response predictions (Jiang et al., 2018). Differences in response rates between high– and low–CAF-risk groups were examined by the chi-squared test, and the predictive efficacy of the CAF risk signature was evaluated by ROC curves and area under the curve (AUC) values.

### Somatic Alteration Data Collection and Analyses

The somatic mutation data of the TCGA-STAD cohort were downloaded *via* the GDCquery_Maf() function (pipelines = “mutect2” (Cibulskis et al., 2013)) of TCGAbiolinks R package (Colaprico et al., 2016). The top 20 highest mutational frequencies in both low– and high–CAF-risk groups were recognized and visualized *via* maftools R package (Mayakonda et al., 2018). Tumor mutation burden (TMB) has been proposed as an immunotherapy efficacy predictor (Yarchoan et al., 2017), and the TMB value of each STAD sample was then calculated *via* the tmb() function of maftools package, and Spearman’s correlation between TMB and CAF risk scores were analyzed.

### Enrichment Analyses

Gene set enrichment analysis (GSEA) was performed to explore the enriched hallmark and KEGG pathway gene sets between high– and low–CAF-risk groups in GSE84437 *via* enrichplot and clusterProfiler R packages. The “c2. cp.kegg.v7.4. symbols” and “h.all.v7.4. symbols” gene sets were derived from the Molecular Signatures Database (MSigDB) (Liberzon et al., 2015). Furthermore, ssGSEA was applied to calculate the enrichment scores of EMT, TGF-β, and angiogenesis hallmark gene sets (Hänzelmann et al., 2013). Spearman’s correlations analysis was performed to assess the correlation between the CAF risk score and gene set enrichment scores.

### Validation *via* Cancer Cell Line Encyclopedia (CCLE) and Human Protein Atlas (HPA) Databases

For cellular level validation, the mRNA expressions of the identified markers in 38 fibroblasts and 39 GC cell lines were downloaded from the CCLE database (https://portals.broadinstitute.org/ccle) (Ghandi et al., 2019), and we examined their expression patterns in fibroblasts and CRC cell lines *via* heat map and Wilcoxon tests. In addition, with respect to protein level investigation, immunohistochemical (IHC) staining images of these markers in GC tissues were downloaded from the HPA online database (https://www.proteinatlas.org/) (Uhlén et al., 2015), and the target protein localization could be directly observed.

### Statistical Analysis

All statistical analyses were performed using R software (version 3.6.3; https://www.r-project.org/). The median CAF risk score was the cutoff value for each cohort in dividing GC patients into high– and low–CAF-risk subgroups. The Wilcoxon test was applied for pairwise comparisons. The Kaplan–Meier curve with the log-rank test was adopted for overall survival comparisons *via* survival and survminer R packages. *p* < 0.05 was regarded as statistically significant.

## Results

### Higher CAF Infiltrations and Stromal Scores Indicate Worse OS in GC Patients

**FIGURE 1:**
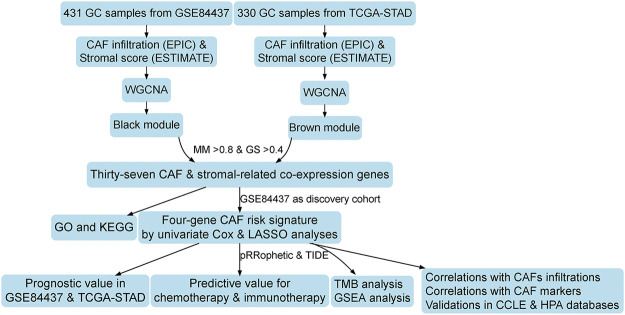
Work flow of this study.

**FIGURE 2:**
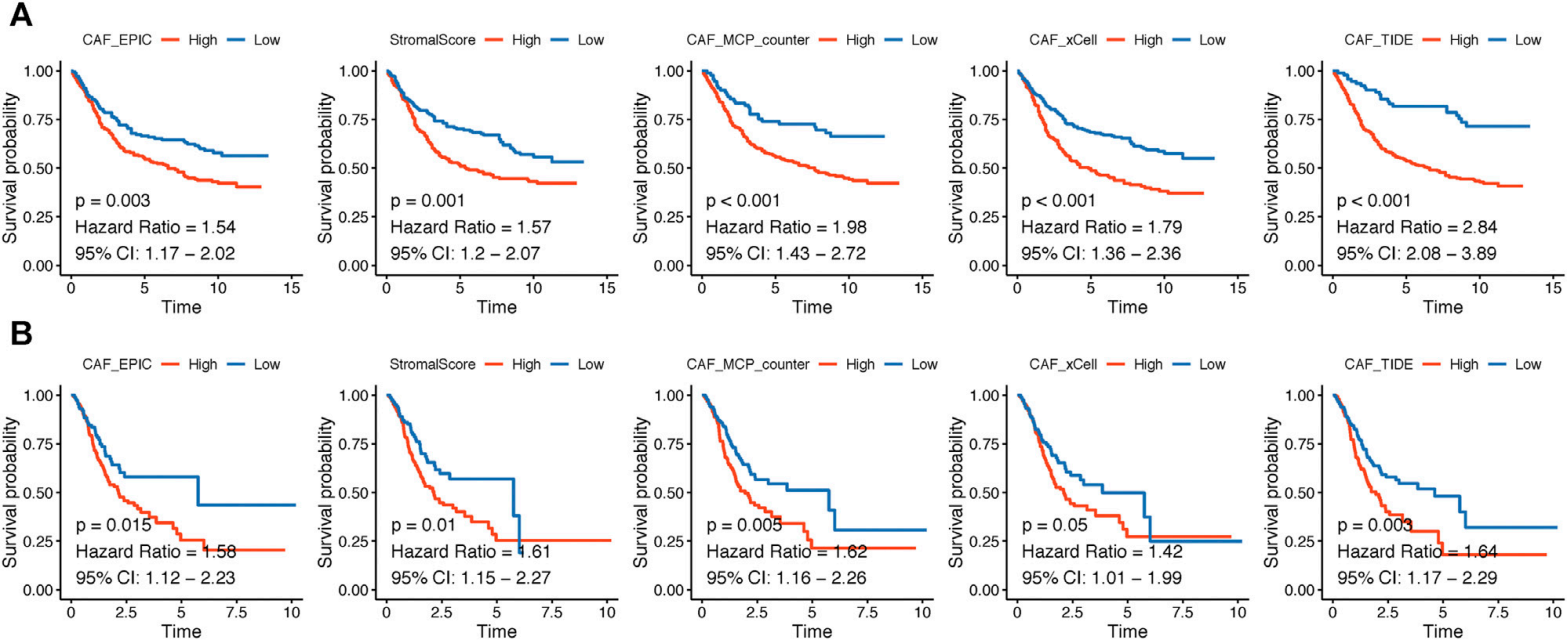
**(A–B).** Kaplan–Meier analyses showing gastric cancer patients with higher CAF infiltrations as well as stromal scores had worse overall survival in GSE84437 **(A)** and TCGA-STAD **(B)**.

The flowchart of this research is displayed in Figure 1. CAF infiltrations were multiply predicted by EPIC, xCell, MCP-counter, and TIDE methods, and the stromal score was calculated by the estimate algorithm. Their prognostic values on OS were evaluated *via* log-rank tests; Kaplan–Meier curves indicated that higher CAF infiltrations and stromal scores were notably correlated with poorer OS of GC patients in both GSE84437 (Figure 2A) and TCGA-STAD (Figure 2B) cohorts, which highlighted the importance of further exploration of CAF and stromal-related genes for GC. Herein, the EPIC-estimated CAF abundances and stromal scores were summarized as phenotype data for the subsequent analysis, and the other three estimated CAF infiltrations data were utilized for the identified CAF model external validations.

### Co-Expression Network of CAF and Stromal Scores

**FIGURE 3:**
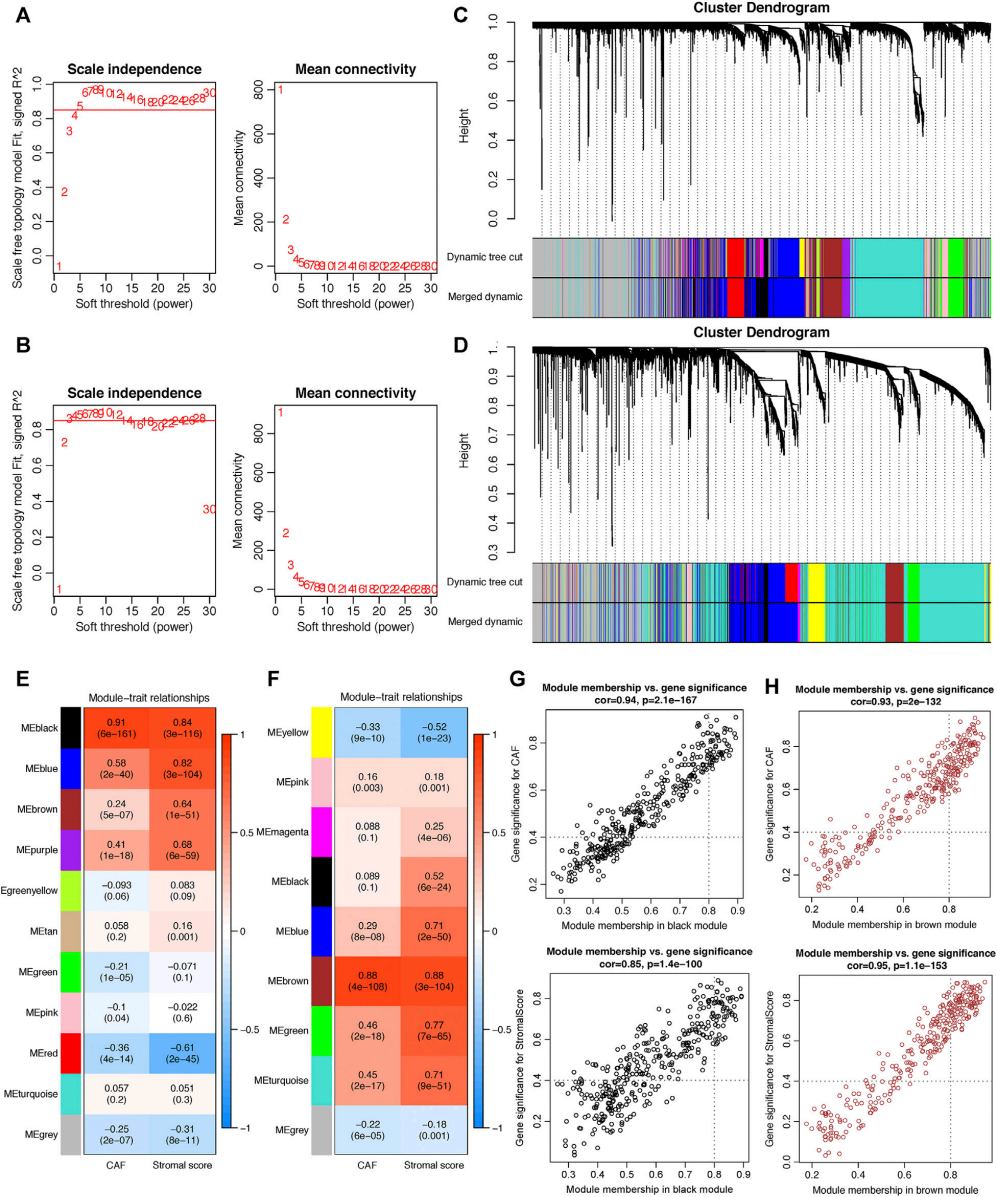
Co-expression network constructed by WGCNA. **(A–B).** The soft-thresholding power (β) of 8 and 6 was, respectively, selected based on the scale-free topology criterion in GSE84437 **(A)** and TCGA-STAD **(B)**. **(C–D).** Clustering dendrograms showing genes with similar expression patterns were clustered into co-expression modules in GSE84437 **(C)** and TCGA-STAD **(D)**. The gray module indicates that genes were not assigned to any module. **(E–F)** Module-trait relationships revealing the correlations between each gene module eigengene and phenotype in GSE84437 **(E)** and TCGA-STAD **(F)**. **(G–H)** Scatter plots of the module membership (MM) and gene significance (GS) of each gene in the black module of GSE84437 **(G)** and the brown module of TCGA-STAD **(H)**. The horizontal axis is the correlation between the gene and co-expression module, and the vertical axis is the correlation between the gene and phenotype.

WGCNA analysis was performed in both GSE84437 and TCGA-STAD. To construct a scale-free topology network, the soft threshold power (β) of 8 in GSE84437 (scale-free *R* ^2^ = 0.97) (Figure 3A) and 6 in TCGA-STAD (scale-free *R* ^2^ = 0.87) (Figure 3B) was estimated. For GSE84437, the hierarchical clustering tree revealed that 11 co-expression models were clustered (Figure 3C), and the black module had the strongest positive correlation with the CAF proportion (Cor = 0.91, P = 6e-161) and stromal score (Cor = 0.84, P = 3e-116) (Figure 3E). For TCGA-STAD, the dynamic hybrid cutting clustered 9 co-expression models (Figure 3D), with the brown module having the strongest positive correlation with the CAF proportion (Cor = 0.88, P = 4e-108) and stromal score (Cor = 0.88, P = 3e-104) (Figure 3F). Hence, the two modules were focused for in-depth investigations. A total of 356 and 302 genes were incorporated in the black and brown modules, respectively. In the black module, the scatter plots illustrated the strong correlations between MM and GS for CAF (Cor = 0.94, *p* = 2.1e-167) and stromal scores (Cor = 0.85, *p* = 1.4e-100) (Figure 3G); in the brown module, the strong correlations were also observed between MM and GS for CAF (Cor = 0.93, P = 2e-132) and stromal scores (Cor = 0.95, *p* = 1.1e-153) (Figure 3H). Then, by taking MM > 0.8 and GS > 0.4 as the threshold criteria, a total of 48 genes in the black model of GSE84437 and 101 genes in the brown module of TCGA-STAD were, respectively, screened out as hub genes which are highly correlated with CAF and stromal scores.

### Functional Analyses of Hub Genes

**FIGURE 4:**
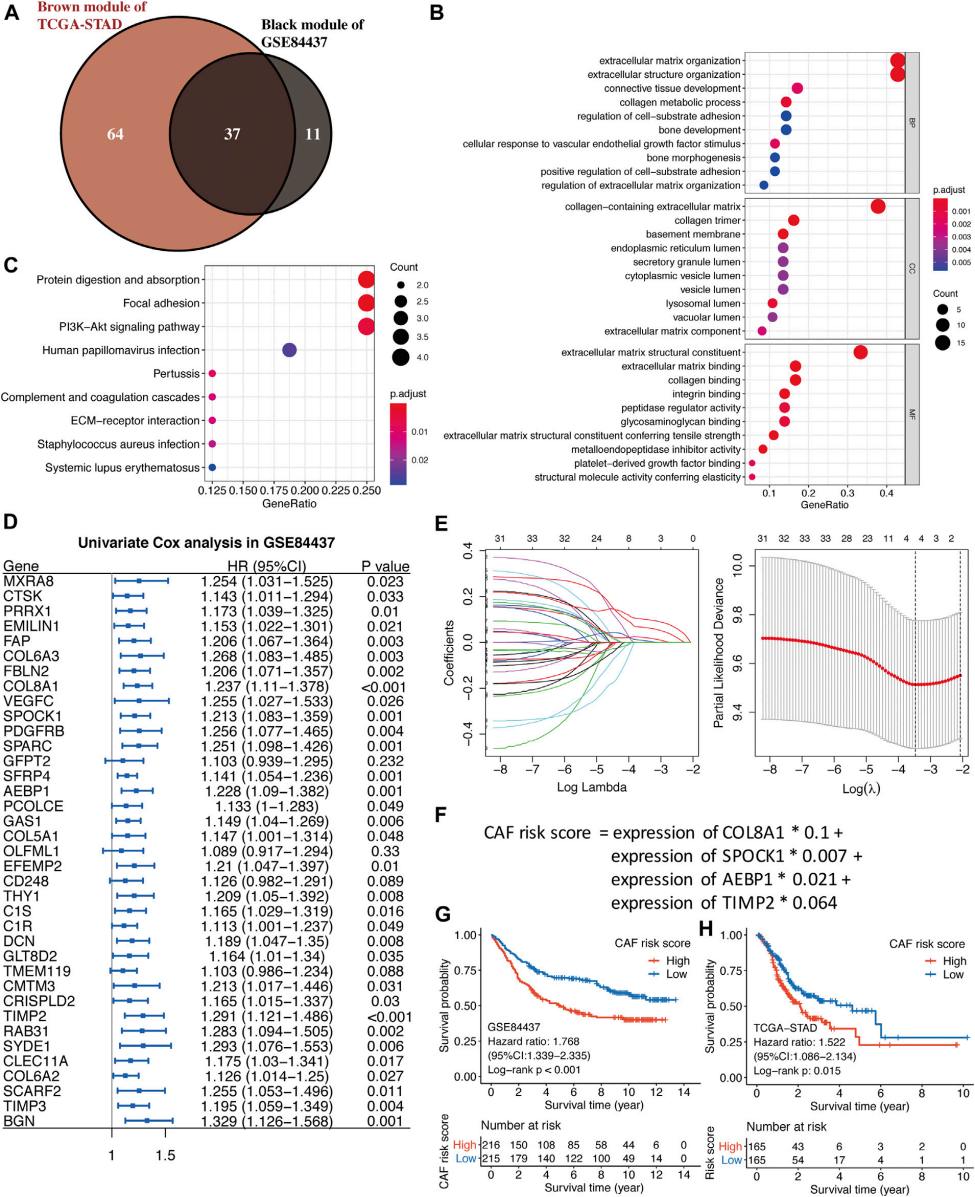
**(A)** The intersection of GSE84437 black and TCGA-STAD brown module genes was presented in the Venn diagram. **(B–C)** GO analyses of the enriched biological process (BP), cellular component (CC), and molecular function (MF) terms **(B)** and KEGG pathway analysis **(C)** of the 37 genes. **(D)** Univariate Cox analysis for the screening of overall survival-associated genes in GSE84437. **(E)** Coefficient profiles of least absolute shrinkage and selection operator (LASSO) Cox regression analysis, and the adjustment parameter (lambda) was calculated based on the partial likelihood deviance with ten-fold cross validation. **(F)** Formulation of the CAF risk model. **(G,H)** Kaplan–Meier analyses identified gastric cancer patients in the high–CAF-risk group which exhibited worse overall survival in both GSE84437 **(G)** and TCGA-STAD **(H)** cohorts.

As shown in Figure 4A, by the intersection of two hub gene sets, 37 common genes were detected and visualized *via* a Venn diagram. Subsequently, we performed GO and KEGG analyses on these 37 genes. Extracellular matrix organization and extracellular structure organization were the major enriched BP terms; the collagen-containing extracellular matrix and extracellular matrix structural constituents were the major enriched CC and MF terms, respectively. Protein digestion and absorption, focal adhesion, and the PI3K-Akt signaling pathway were the main enriched KEGG pathways.

### Construction of the Stromal CAF-Based Prognostic Risk Model

Four hundred and thirty-one GC samples from GSE84437 were used as the training cohort owing to the larger sample size, and 330 TCGA-STAD samples were used as the validation group. By performing univariate Cox regression analysis of the 37 common hub genes, 33 OS-related genes with *p* < 0.05 were screened out and subjected to the following LASSO Cox regression analysis (Figures 4D,E). Four genes were finally identified for the CAF risk model construction: CAF risk score = expression of COL8A1 * 0.1 + expression of SPOCK1 * 0.007 + expression of AEBP1 * 0.021 + expression of TIMP2 * 0.064 (Figure 4F). GC patients in each cohort were divided into high– and low–CAF-risk groups with the median risk score as the cutoff value. Kaplan–Meier curves revealed that GC patients in the high–CAF-risk group experienced worse OS than those in the low–CAF-risk group in both GSE84437 (HR = 1.768, 95%CI: 1.339–2.335, log-rank *p* < 0.001) (Figure 4G) and TCGA-STAD (HR = 1.522, 95%CI: 1.086–2.134, log-rank *p* = 0.015) (Figure 4H). These results indicated CAF and stromal-related signature genes were crucial prognostic markers in GC.

### CAF Signature Genes Were Highly Correlated With CAF Infiltrations and CAF Markers

**FIGURE 5:**
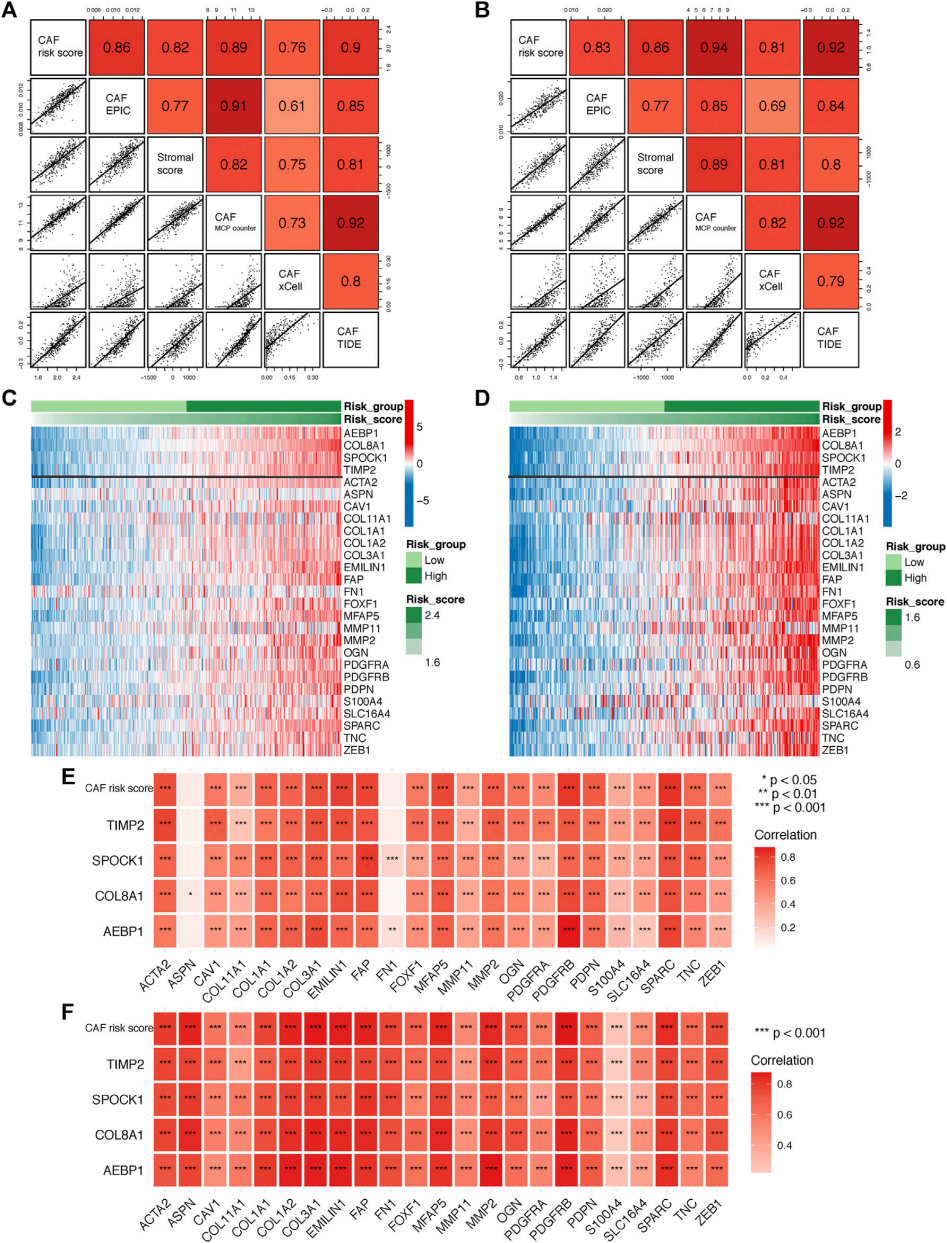
**(A–B)** Spearman’s correlation analyses revealing the CAF risk score was strongly and positively correlated with stromal scores and multi-estimated CAF infiltrations in GSE84437 **(A)** and TCGA-STAD **(B)** cohorts. **(C–D)** The heat map revealing the expression patterns of CAF markers identified four CAF genes with the CAF risk score in GSE84437 **(C)** and TCGA-STAD **(D)** cohorts. **(E–F)** The CAF risk score and four signature genes were positively correlated with literature that reported CAF markers in GSE84437 **(E)** and TCGA-STAD **(F)** cohorts.

To further verify the robustness of the CAF model as an indicator in predicting CAF infiltrations, we performed Spearman’s correlation analyses between the CAF risk score and stromal score as well as CAF abundances predicted by EPIC and other three methods: xCell, MCP-counter, and TIDE. Consistently, we observed the CAF risk score was strongly and positively correlated with multi-estimated CAF infiltrations and the stromal score in both GSE84437 (Figure 5A) and TCGA-STAD (Figure 5B) cohorts. Moreover, we observed the CAF risk score and the expression levels of the four genes were highly and positively correlated with a bunch of the collected CAF markers in both GSE84437 (Figures 5C,E) and TCGA-STAD (Figures 5D,F) cohorts.

### Sensitivity of Chemotherapy and Immunotherapy Between CAF-Risk Groups

**FIGURE 6:**
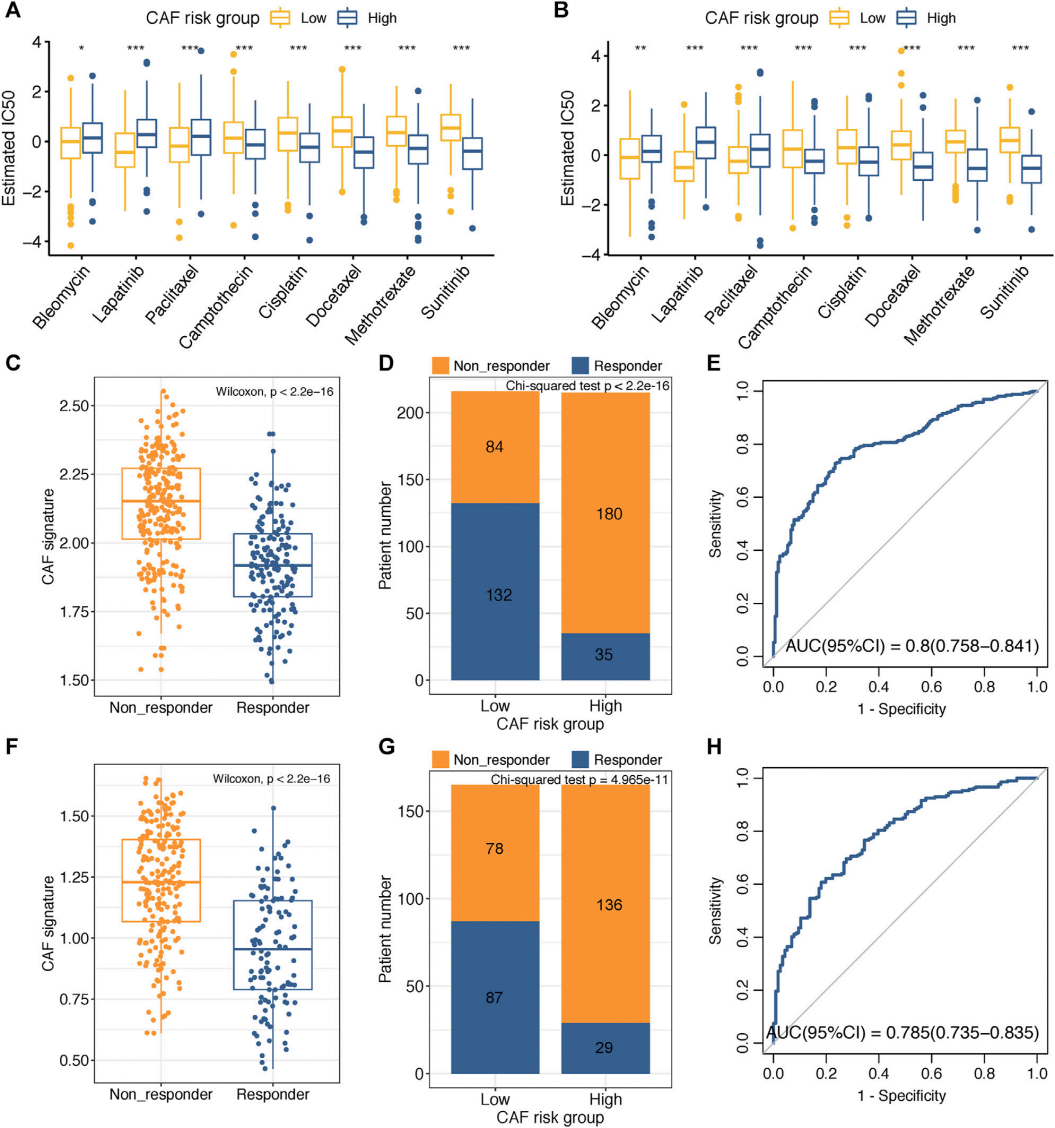
**(A–B)** Box plots comparing IC50 values of several chemotherapy drugs between high– and low–CAF-risk groups in GSE84437 **(A)** and TCGA-STAD **(B)** cohorts. **(C–H)** TIDE immunotherapy prediction analyses. **(C,F)** The CAF risk score between TIDE-predicted immunotherapy-responders and non-responders in GSE84437 **(C)** and TCGA-STAD **(F)**; **(D,G)** Distributions of responders and non-responders in high– and low– CAF-risk groups in GSE84437 **(D)** and TCGA-STAD **(G)**; **(E,H)** ROC curves of the CAF risk score in predicting immunotherapy responses in GSE84437 **(E)** and TCGA-STAD **(H)**. ∗*p* < 0.05, ∗∗*p* < 0.01, ∗∗∗*p* < 0.001.

Adjuvant chemotherapy following radical surgery has been the standard approach regarding GC. IC50 values of several drugs mentioned in the methods section were estimated based on the GDSC database. Wilcoxon analyses identified significant differences in IC50 values between GC patients in high– and low–CAF-risk groups, with high–CAF-risk GC patients revealing increased sensitivity to camptothecin, cisplatin, docetaxel, methotrexate, and sunitinib, while the low-CAF score subgroup was estimated to be more sensitive to bleomycin, lapatinib, and paclitaxel in both GSE84437 (Figure 6A) and TCGA-STAD (Figure 6B) cohorts.

Immunotherapy using immune checkpoint inhibitors has brought hope to GC patients. We applied the TIDE method to assess whether the CAF risk score could serve as an immunotherapy predictor for GC patients. For GSE84437, the CAF score in the non-responder subgroup (*n* = 264) was significantly higher than that in the responder cohort (*n* = 167) (Wilcoxon test, *p* < 2.2e-16; Figure 6C); higher sensitivity to immunotherapy was observed for GC patients in the low–CAF-risk group (132/216) than that in the high–CAF-risk group (35/215) (Chi-square test, *p* < 2.2e-16; Figure 6D). For TCGA-STAD, the CAF score was also significantly higher in the non-responder subgroup (*n* = 214) than that in the responder cohort (*n* = 116) (Wilcoxon test, *p* < 2.2e-16; Figure 6F); GC patients in the low–CAF-risk group were much more sensitive to immunotherapy (87/165) than those in high-CAF risk group (29/165) (chi-square test, *p* < 2.2e-16; Figure 6G). Furthermore, the AUC values of 0.8 (95%CI: 0.758–0.841) in GSE84437 (Figure 6E) and 0.785 (95%CI: 0.735–0.835) in TCGA-STAD (Figure 6H) indicated the excellent performance of our CAF model for immunotherapy response predictions.

### Correlation Between CAF-Related Signature and Somatic Variation

**FIGURE 7:**
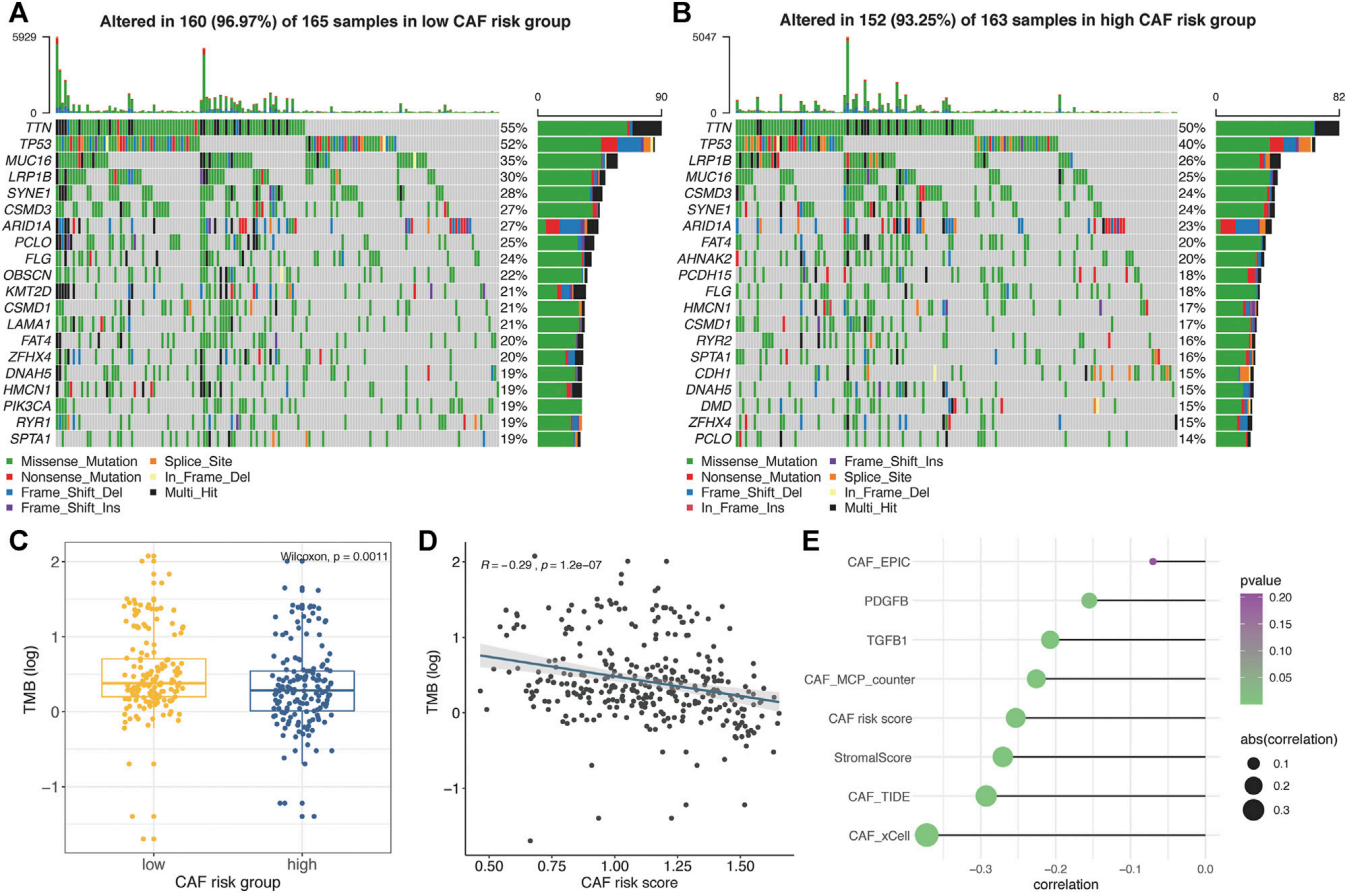
**(A–B)** Oncoplots depicting the top 20 mutational genes in low– **(A)** and high– **(B)** CAF-risk groups of TCGA-STAD. **(C)** The boxplot revealing TMB values was higher in the low–CAF-risk group. **(D)** Spearman’s correlation analyses revealed that the CAF risk score was significantly and negatively correlated with the TMB value. **(E)** Spearman’s correlation analyses revealed the TMB value was negatively correlated with CAF activators as well as CAF infiltrations.

The top 20 genes with highest mutational frequencies in the low– (Figure 7A) and high– (Figure 7B) CAF-risk subgroups were, respectively, recognized and displayed as waterfall plots. Intriguingly, several frequent mutational genes were shared in both low– and high–CAF-risk groups, including TTN, TP53, MUC16, LRP1B, SYNE1, CSMD3, ARID1A, PCLO, FLG, CSMD1, FAT4, ZFHX4, DNAH5, HMCN1, and SPTA1. In addition, mutations of LAMA1, RYR1, OBSCN, KMT2D, and PIK3CA genes were more common in the low–CAF-risk group, while mutations of DMD, AHNAK2, PCDH15, RYR2, and CDH1 belonged specifically to the top 20 frequent mutational genes in the high–CAF-risk group. Subsequently, we observed that the TMB values were significantly higher in the low–CAF-score subgroup (Wilcoxon test, *p* = 0.0011, Figure 7C), and Spearman’s correlation analysis revealed that the CAF-risk score was significantly and negatively correlated with the TMB value (Cor = -0.29, *p* = 1.2e-07, Figure 7D). Furthermore, Spearman correlation analyses also confirmed that the TMB values were negatively correlated with stromal CAF infiltrations as well as CAF-activating factors like TGF-β (Quante et al., 2011) and PDGF (Pietras et al., 2008) (Figure 7E), suggesting that higher TMB might be also able to intense tumor-killing effects *via* modulating a stromal fibroblast-weak local microenvironment.

### GSEA of the Four-Gene CAF Signature

**FIGURE 8:**
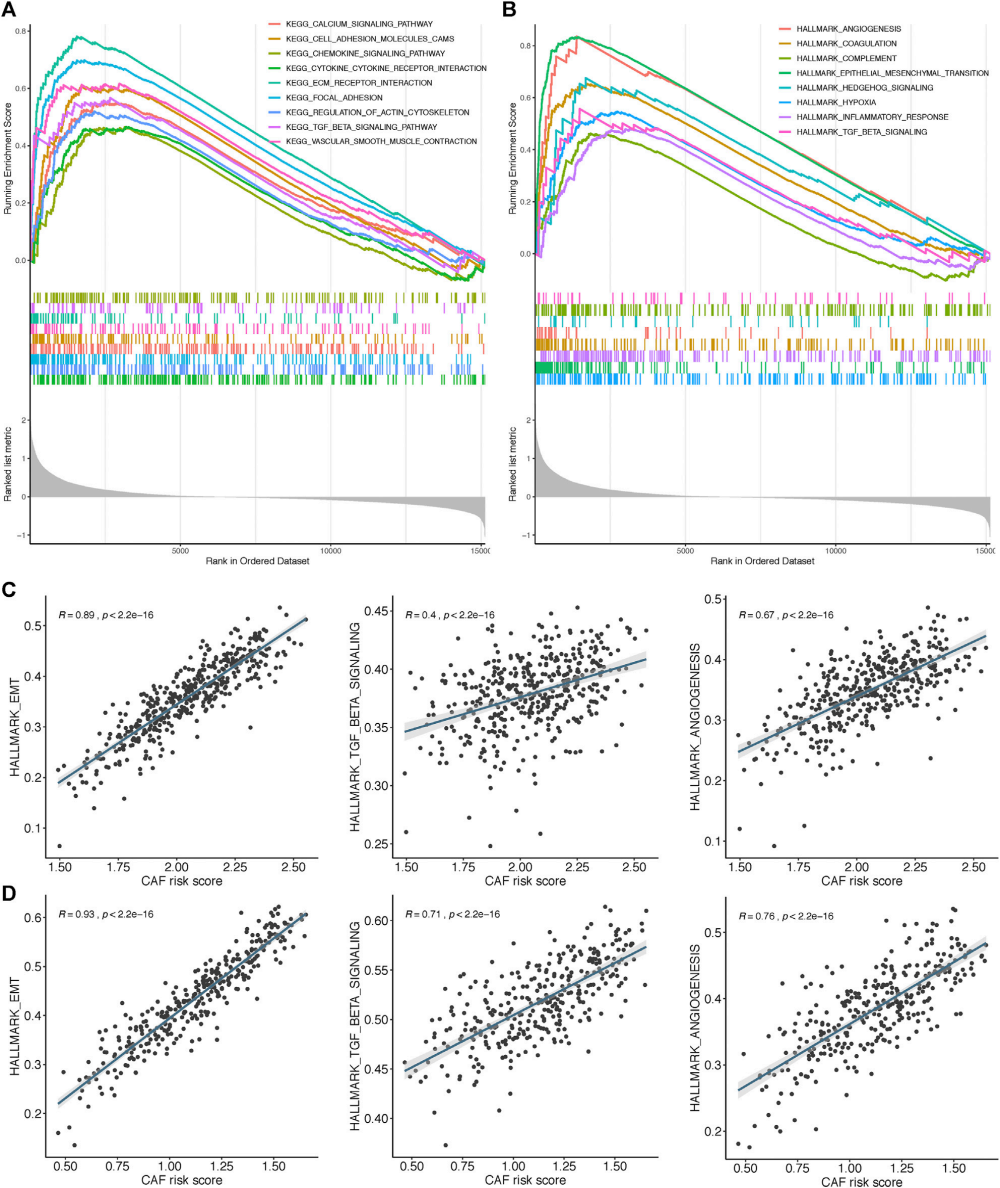
Gene set enrichment analysis (GSEA) of KEGG **(A)** and hallmark **(B)** gene sets between high‐and low‐CAF risk groups. **(C,D)** ssGSEA results showed CAF risk score was positively correlated with EMT, TGF‐β and angiogenesis enrichment scores in both GSE84437 **(C)** and TCGA‐STAD **(D)**.

To further elucidate the functional enrichment of the CAF signature, GSEA was performed on the GSE84437 dataset between high– and low–CAF-risk groups. As displayed in Figure 8A, the major enriched KEGG signaling pathways were calcium signaling pathway, ECM receptor interaction, chemokine signaling, and transforming growth factor beta (TGF-β) signaling pathways. Additionally, genes in the high–CAF-risk group were mainly enriched in angiogenesis, epithelial–mesenchymal transition (EMT), inflammatory response, and TGF-β signaling hallmarker gene sets (Figure 8B). Extensively, ssGSEA results also showed the CAF risk score was positively correlated with EMT, TGF-β, and angiogenesis enrichment scores in both GSE84437 (Figure 8C) and TCGA-STAD (Figure 8D).

### Multidimensional Validation of Key Genes in CCLE and HPA Databases

**FIGURE 9:**
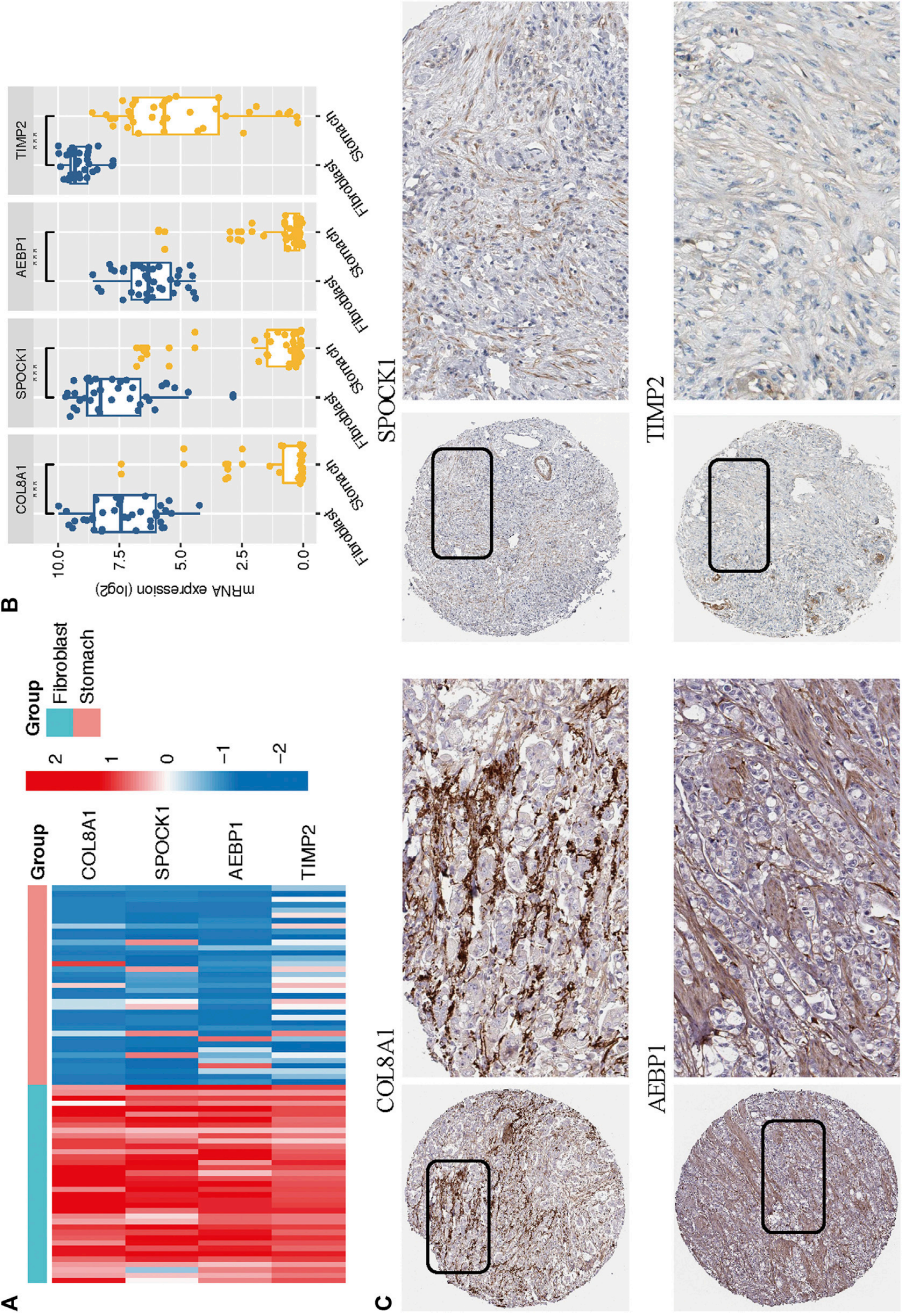
**(A–B)** The mRNA expression levels of the four CAF genes in the fibroblasts and gastric cancer cell lines were illustrated in the heat map **(A)** and compared by Wilcoxon analysis **(B)**. **(C)** Protein expressions of COL8A1, SPOCK1, AEBP1, and TIMP2 in gastric cancer specimens from the Human Protein Atlas database.

Based on the CCLE database, we verified that the mRNA expressions of the four hub genes (COL8A1, SPOCK1, AEBP1, and TIMP2) were higher in fibroblast cell lines than those in GC cell lines (Wilcoxon test, all *p* < 0.001; Figures 9A,B). In addition, to determine the protein expression characteristics of these CAF signature genes, we analyzed the IHC images from the HPA database. The data demonstrated that these proteins were deeply stained in GC stroma (Figure 9C). These verifications implied that these four genes might be CAF-specific markers.

## Discussion

Gastric cancers, especially poorly and undifferentiated gastric cancers, often exhibit massive fibrosis with abundant infiltration of CAFs, which shield TME from antitumor lymphocyte infiltrations and contribute to GC progression, treatment resistance, and immunosuppression (Abe et al., 2017; Ham et al., 2019). Consistently, we observed that higher CAF and stromal scores were associated with worse OS after initial treatment in GC. Therefore, investigation of novel molecular targets in GC is pivotal for the development of stromal CAF-targeting therapies. This is the first research based on WGCNA and multiple computational algorithms to mine the mutual CAF and stromal co-expressed networks in 2 GC cohorts: GSE84437 and TCGA-STAD. By applying univariate Cox and LASSO regression algorithms, a four-gene (COL8A1, SPOCK1, AEBP1, and TIMP2) prognostic CAF model was constructed and validated. By taking the median CAF risk score as the cutoff value, we observed high–CAF-risk GC patients were more sensitive to camptothecin, cisplatin, docetaxel, methotrexate, and sunitinib. In addition, based on the TIDE online algorithm, we observed the lower CAF risk score was highly correlated with improved immunotherapeutic effects in GC patients, and higher TMB levels were observed in low–CAF-risk group STAD patients, which indicated that the CAF model could potentially serve as an immunotherapeutic stratification biomarker for GC. However, interactions between TMB and CAF infiltrations have not been well studied to date. Our study first revealed that the TMB levels were also negatively associated with CAF activators as well as infiltrations in GC patients. It is widely acknowledged that cancer cells with a high level of mutations are easier to be recognized by the immune system, which can then strengthen the immune response and lead to improved immunotherapeutic efficacy (Miao et al., 2018). Based on our analyses, we propose another mechanism that higher TMB might also be able to intense tumor-killing effects *via* modulating a stromal fibroblast-weak local microenvironment. However, more experiments are needed to clarify the crosstalk between CAFs and TMB.

GSEA revealed that EMT, TGF-β signaling, hypoxia, and angiogenesis gene sets were highly and significantly enriched in the high–CAF-risk group; ssGSEA results also showed that the CAF risk score was positively correlated with EMT, TGF-β, and angiogenesis-enrichment scores in both two cohorts. Polarized epithelial cells gain invasive capacities through the EMT process (Huang et al., 2015), and TGF-β signaling has been reported to be responsible for the CAF activation (Yeung et al., 2013; Zheng et al., 2016; Ishimoto et al., 2017). Interactively, CAFs are capable of synergistically initiating and enhancing EMT (Bhowmick et al., 2004; Lee et al., 2006; Thiery and Sleeman, 2006; Ham et al., 2019); CAFs could also regulate and maintain the stemness of gastric cancer cells *via* TGFβ signaling (Hasegawa et al., 2014). Pathological angiogenesis has been widely described as a crucial process enabling the expansion of cancerous tissues, as well as the invasion and metastasis of GC cells (Chen et al., 2004; Hoff and Machado, 2012; Forma et al., 2021). CAFs contributed dominantly to the uncontrolled angiogenesis by inducing a hypoxia TME (Kugeratski et al., 2019) and producing pro-angiogenic factors like galectin-1 (Tang et al., 2016), vascular endothelial growth factor (VEGF) (De Francesco et al., 2013), and hepatocyte growth factor (HGF) (Ding et al., 2018).

To guarantee the model’s robustness and avoid over-fitting, we adopted four bioinformatics methods to quantify CAF infiltrations in GC: the EPIC method for model construction and xCell, MCP-counter, and TIDE methods for correlation verifications, and we found that our model was strongly correlated with CAF infiltrations as well as CAF markers. Meanwhile, according to the CCLE database, we further confirmed that the expressions of four identified genes were significantly higher in fibroblast cell lines, and IHC images from the HPA database also revealed higher staining of these proteins in stromal parts of GC. These results implied these genes as CAF-specific markers for GC, and our model was capable of accurately assessing CAF infiltration levels.

With respect to the four identified markers in the model, elevated expression of COL8A1 has been found in CAFs and was significantly associated with a high risk of death in head and neck squamous cell carcinoma (Lai et al., 2019). Zhang et al. identified COL8A1 as the prognostic hub gene highly correlated with Wnt2, which is elevated selectively in CAFs, and high co-expression of COL8A1 and Wnt2 was an independent adverse prognostic factor for colon cancer patients (Katoh, 2001; Zhang et al., 2020). At an epithelial cellular level, Zhou et al. reported that the knockdown of COL8A1 significantly suppressed the proliferation and promoted the apoptosis of GC cells (Zhou et al., 2020). As for SPOCK1, studies have proved SPOCK1 as an EMT-related marker that was closely correlated with tumorigenesis and invasiveness in gastric cancer (Yan et al., 2017; Chen et al., 2018), prostate cancer (Wang et al., 2016), pancreatic cancer (Li et al., 2020), gallbladder cancer (Shu et al., 2015), and lung cancer (Miao et al., 2013). We observed that high–CAF-risk group GC patients were less sensitive to several drugs like lapatinib, and this result fits well with the finding that SPOCK1-regulated EMT derived the acquiring of lapatinib resistance in HER2-positive GC (Kim et al., 2014). Veenstra et al. identified that SPOCK1 expressed restrictively in tumor stroma, and the stromal SPOCK1 would promote pancreatic ductal adenocarcinoma invasion by mediating extracellular matrix remodeling (Veenstra et al., 2017). Sasaki et al. identified AEBP1 as a novel CAF- and EMT-related protein responsible for tumor invasiveness and metastasis in basal cell carcinoma, squamous cell carcinoma, and malignant melanoma (Sasaki et al., 2018). AEBP1 has also been reported as a pivotal proinflammatory mediator (Majdalawieh et al., 2006; Majdalawieh et al., 2007; Majdalawieh and Ro, 2010), and the overexpression of stromal AEBP1 would induce mammary tumorigenesis *via* paracrine proinflammatory signaling (Holloway et al., 2012). In addition, AEBP1 is upregulated in vascular endothelial cells and promotes tumor angiogenesis in colorectal cancer by inducing angiogenesis-related genes like AQP1 (Yorozu et al., 2020). AEBP1 has also been demonstrated as an adverse prognostic marker in GC that facilitates invasion and migration, metastasis, and EMT of GC cells *via* activating NF-κB signaling (Liu et al., 2018). TIMP2 was previously found as a matrix metalloproteinase (MMP) inhibitor (Basu et al., 2012) that restrained cell proliferation and metastasis in GC (Johansson et al., 2010) and breast cancer (Mendes et al., 2007). However, literature on its functions in cancer is inconsistent. TIMP2 was correlated with higher pN and pM stages as well as unfavorable prognosis in GC (Alakus et al., 2010; Wang et al., 2018). Besides, TIMP2 expressed by CAFs was independently related to lower relapse-free and overall survival in breast cancer (Eiró et al., 2015; Cid et al., 2018). Nonetheless, not much of their functions are known in CAFs of GC, which necessitates further experiments of the four CAF marker’s mechanisms that underline the invasiveness and metastasis, drug resistance, and immunosuppression of GC.

Some limitations should be noted in our research. First, this was a retrospective bioinformatic analysis based on two public gene expression data; the real prognostic and therapeutic values of the CAF model should be cross-validated in multicenter and perspective data. In addition, the specific biological roles of the CAF signature biomarkers in GC should be verified through molecular and animal experiments. Nevertheless, our instructive results could serve as a framework for the future mapping of CAFs in GC.

## Conclusion

In summary, we confirmed higher infiltration of stromal CAFs in TME was correlated with worse prognosis in GC and identified COL8A1, SPOCK1, AEBP1, and TIMP2 as novel prognostic CAF biomarkers by constructing an integrated co-expression network of infiltrated CAF and stromal scores. A CAF model comprising of these four markers was constructed and validated, which could precisely predict prognosis, CAF infiltrations, chemotherapy, and immunotherapy responses in GC patients, which might provide novel insights for targeting CAF therapeutic strategies in GC.

## Data Availability

Publicly available datasets were analyzed in this study. These data can be found here: GDC TCGA Hub of the UCSC XENA database (https://gdc.xenahubs.net); GEO datasets (https://www.ncbi.nlm.nih.gov/geo/): GSE84437.
